# Supplementary material for: Combination Therapy Is Not Associated with Decreased Mortality in Infectious Endocarditis: A Systematic Review and Meta-Analysis
Source: Microorganisms. 2024 Nov 2;12(11):2226. doi: 10.3390/microorganisms12112226 (PMC11596167; doi:10.3390/microorganisms12112226)

Supplemental Content

Combination therapy not associated with decreased mortality in infectious endocarditis: A systematic review and meta-analysis

Appendix S1. Search Strategy report: original search

Librarian searcher: Sarah Cantrell, MLIS; Duke University Medical Center Library & Archives, Duke University School of Medicine

Peer-review of search conducted by: Steph Hendren, MLIS; Duke University Medical Center Library & Archives, Duke University School of Medicine

Database: MEDLINE (via Ovid)
Search date: 7/28/2023 *Ovid MEDLINE® ALL 1946 to July 27, 2023*
Search update: 7/29/2024 *Ovid MEDLINE® ALL 1946 to July 26, 2024*

Search Set + Description	Search Strategy	Results 7/28/23	Results 7/29/24
#1 *Infective endocarditis terms*	exp endocarditis/ or endocardit*.ti,ab.	44910	46498
#2 *ABX terms*	exp anti-bacterial agents/ or exp nafcillin/ or exp oxacillin/ or exp penicillins/ or exp ampicillin/ or exp methicillin/ or exp floxacillin/ or exp amoxicillin/ or exp piperacillin/ or exp "piperacillin, tazobactam drug combination"/ or exp cefazolin/ or exp cefepime/ or exp cefotaxime/ or exp ceftazidime/ or exp ceftriaxone/ or exp gentamicins/ or exp streptomycin/ or exp tobramycin/ or exp daptomycin/ or exp vancomycin/ or exp linezolid/ or exp rifampin/ or exp ertapenem/ or exp imipenem/ or exp meropenem/ or exp ciprofloxacin/ or exp beta-lactams/ or exp cephalosporins/ or exp carbapenems/ or exp aminoglycosides/ or exp fluoroquinolones/ or (antibiotic* or anti-biotic* or antiinfective* or anti-infective* or antibacter* or anti-bacter* or nafcillin or oxacillin or penicillin or penicillins or ampicillin or methicillin or flucloxacillin or amoxicillin or piperacillin or piperacillin-tazobactam or cefazolin or cefepime or cefotaxime or ceftaroline or ceftazidime or ceftriaxone or gentamicin or gentamicins or streptomycin or tobramycin or daptomycin or vancomycin or dalbavancin or linezolid or rifampin or ertapenem or imipenem or meropenem or ciprofloxacin or beta-lactam or beta-lactams or cephalosporin or cephalosporins or carbapenem or carbapenems or aminoglycoside or aminoglycosides or fluoroquinolone or fluoroquinolones).ti,ab.	1193818	1245110
#3 *Combo Tx terms*	drug therapy, combination/ or (combination or combinations or combined or combining or combo or combos or polytherap* or multimodal or multi-modal or "add on" or add-on or co-treat* or cotreat* or co-therap* or	2865137	3065391

	cotherap* or co-admin* or coadmin* or synerg*).ti,ab. or (plus or multi or multiple or dual).ti.		
#4 combining	1 and 2 and 3	2520	2610
#5	4 not ("case report".ti. or "case reports".ti. or comment.pt. or congress.pt. or editorial.pt.)	2312	2392
#6 *Search update*	Limit 5 to da=20230701-20241231	n/a	89
Exemplar test to ensure search captures all of these studies	(5 or 6) and (32725216 OR 434682 OR 1929035 OR 29408610 OR 8749624).ui.	5/5	5/5

Database: Embase (via Elsevier)
Search date: 7/28/2023
Search update: 7/29/2024
Note: Search from the Results page

Search Set + Description	Search Strategy	Results 7/28/23	Results 7/29/24
#1 *Infective endocarditis terms*	'endocarditis'/exp OR endocardit*:ti,ab	72814	76497
#2 *ABX terms*	'antibiotic agent'/exp OR (antibiotic* OR antibiotic* OR antiinfective* OR anti?infective* OR antibacter* OR anti?bacter* OR nafcillin OR oxacillin OR penicillin OR penicillins OR ampicillin OR methicillin OR flucloxacillin OR amoxicillin OR piperacillin OR piperacillin?tazobactam OR cefazolin OR cefepime OR cefotaxime OR ceftaroline OR ceftazidime OR ceftriaxone OR gentamicin OR gentamicins OR streptomycin OR tobramycin OR daptomycin OR vancomycin OR dalbavancin OR linezolid OR rifampin OR ertapenem OR imipenem OR meropenem OR ciprofloxacin OR beta?lactam OR beta?lactams OR cephalosporin OR cephalosporins OR carbapenem OR carbapenems OR aminoglycoside OR aminoglycosides OR fluoroquinolone OR fluoroquinolones):ti,ab	2258488	2380045
#3 *Combo Tx terms*	'drug combination'/de OR (combination OR combinations OR combined OR combining OR combo OR combos OR polytherap* OR multimodal OR multi?modal OR 'add on' OR add?on OR co?treat* OR cotreat* OR co?therap* OR cotherap* OR co?admin* OR coadmin* OR synerg*):ti,ab OR (plus OR multi OR multiple OR dual):ti	3800742	4069134
#4 combining	#1 AND #2 AND #3	4060	4378
#5	#4 NOT ('case report':ti OR 'case reports':ti OR 'case study':ti OR 'case studies':ti OR 'editorial'/exp OR [editorial]/lim OR 'letter'/exp OR [letter]/lim OR 'note'/exp OR [note]/lim OR [conference abstract]/lim OR 'conference abstract'/exp OR 'conference abstract'/it)	2771	2910
#6	#5 AND [28-07-2023]/sd	n/a	161

Search update			

Database: Cochrane Central Register of Controlled Trials (via Wiley)
Search date: 7/28/2023
Search update: 7/29/2024
Note: go to Advanced Search > Search Manager

Search Set + Description	Search Strategy	Results 7/28/23	Results 7/29/24
#1 *Infective endocarditis terms*	[mh endocarditis] OR endocardit*:ti,ab	677	724
#2 *ABX terms*	[mh "anti-bacterial agents"] OR (antibiotic*:ti,ab OR anti-biotic*:ti,ab OR anti-infective*:ti,ab OR antibacter*:ti,ab OR anti-bacter*:ti,ab OR nafcillin:ti,ab OR oxacillin:ti,ab OR penicillin:ti,ab OR penicillins:ti,ab OR ampicillin:ti,ab OR methicillin:ti,ab OR flucloxacillin:ti,ab OR amoxicillin:ti,ab OR piperacillin:ti,ab OR piperacillin-tazobactam:ti,ab OR cefazolin:ti,ab OR cefepime:ti,ab OR cefotaxime:ti,ab OR ceftaroline:ti,ab OR ceftazidime:ti,ab OR ceftriaxone:ti,ab OR gentamicin:ti,ab OR gentamicins:ti,ab OR streptomycin:ti,ab OR tobramycin:ti,ab OR daptomycin:ti,ab OR vancomycin:ti,ab OR dalbavancin:ti,ab OR linezolid:ti,ab OR rifampin:ti,ab OR ertapenem:ti,ab OR imipenem:ti,ab OR meropenem:ti,ab OR ciprofloxacin:ti,ab OR beta-lactam:ti,ab OR beta-lactams:ti,ab OR cephalosporin:ti,ab OR cephalosporins:ti,ab OR carbapenem:ti,ab OR carbapenems:ti,ab OR aminoglycoside:ti,ab OR aminoglycosides:ti,ab OR fluoroquinolone:ti,ab OR fluoroquinolones:ti,ab)	53063	56330
#3 *Combo Tx terms*	[mh ^"drug therapy, combination"] OR (combination:ti,ab OR combinations:ti,ab OR combined:ti,ab OR combining:ti,ab OR combo:ti,ab OR combos:ti,ab OR polytherap*:ti,ab OR multimodal:ti,ab OR multi-modal:ti,ab OR "add on":ti,ab OR add-on:ti,ab OR co-treat*:ti,ab OR cotreat*:ti,ab OR co-therap*:ti,ab OR cotherap*:ti,ab OR co-admin*:ti,ab OR coadmin*:ti,ab OR synerg*:ti,ab) OR (plus:ti OR multi:ti OR multiple:ti OR dual:ti)	333179	359793
#4 combining	#1 AND #2 AND #3	73	84
#5 Search update		n/a	8

Database: Web of Science – Science Citation Index Expanded (1900-present), Social Science Citation Index (1900-present), Emerging Sources Citation Index (2019-present) (via Clarivate)
Search date: 7/28/2023
Search update: 7/29/2024
Note: Select indices under Editions; use Advanced Search

Search Set + Description	Search Strategy	Results 7/28/23	7/29/24

#1 *Infective endocarditis terms*	TS=(endocardit*)	40672	44004
#2 *ABX terms*	TS=(antibiotic* OR anti-biotic* OR anti-infective* OR antibacter* OR anti-bacter* OR nafcillin OR oxacillin OR penicillin OR penicillins OR ampicillin OR methicillin OR flucloxacillin OR amoxicillin OR piperacillin OR piperacillin-tazobactam OR cefazolin OR cefepime OR cefotaxime OR ceftaroline OR ceftazidime OR ceftriaxone OR gentamicin OR gentamicins OR streptomycin OR tobramycin OR daptomycin OR vancomycin OR dalbavancin OR linezolid OR rifampin OR ertapenem OR imipenem OR meropenem OR ciprofloxacin OR beta-lactam OR beta-lactams OR cephalosporin OR cephalosporins OR carbapenem OR carbapenems OR aminoglycoside OR aminoglycosides OR fluoroquinolone OR fluoroquinolones)	785488	879548
#3 *Combo Tx terms*	TS=(combination OR combinations OR combined OR combining OR combo OR combos OR polytherap* OR multimodal OR multi-modal OR "add on" OR add-on OR co-treat* OR cotreat* OR co-therap* OR cotherap* OR co-admin* OR coadmin* OR synerg*) OR TI=(plus OR multi OR multiple OR dual)	5111487	5709753
#4 combining	#1 AND #2 AND #3	1826	2127
#5	#4 NOT TI=("case report" OR "case reports" OR "case study" OR "case studies")	1719	2007
#6	Limit 5 to Document Types: Article or Review Article or Proceeding Paper or Early Access	1587	1860
#7 Search update	Limit to Publication Years: 2023 or 2024	n/a	149

Database: CINAHL Complete (via EBSCO)
Search date: 7/28/2023
Search update: 7/29/2024

Search Set + Description	Search Strategy	Results 7/28/23	Results 7/29/24
#1 *Infective endocarditis terms*	(MH "Endocarditis+") OR (TI endocardit* OR AB endocardit*)	7880	8252
#2 *ABX terms*	(MH "Antibiotics+") OR ((TI antibiotic* OR AB antibiotic*) OR (TI anti-biotic* OR AB anti-biotic*) OR (TI antiinfective* OR AB antiinfective*) OR (TI anti-infective* OR AB anti-infective*) OR (TI antibacter* OR AB antibacter*) OR (TI anti-bacter* OR AB anti-bacter*) OR (TI nafcillin OR AB nafcillin) OR (TI oxacillin OR AB oxacillin) OR (TI penicillin OR AB penicillin) OR (TI penicillins OR AB penicillins) OR (TI ampicillin OR AB ampicillin) OR (TI methicillin OR AB methicillin) OR (TI flucloxacillin OR AB flucloxacillin) OR (TI amoxicillin OR AB amoxicillin) OR (TI piperacillin OR	134372	136531

	AB piperacillin) OR (TI piperacillin-tazobactam OR AB piperacillin-tazobactam) OR (TI cefazolin OR AB cefazolin) OR (TI cefepime OR AB cefepime) OR (TI cefotaxime OR AB cefotaxime) OR (TI ceftaroline OR AB ceftaroline) OR (TI ceftazidime OR AB ceftazidime) OR (TI ceftriaxone OR AB ceftriaxone) OR (TI gentamicin OR AB gentamicin) OR (TI gentamicins OR AB gentamicins) OR (TI streptomycin OR AB streptomycin) OR (TI tobramycin OR AB tobramycin) OR (TI daptomycin OR AB daptomycin) OR (TI vancomycin OR AB vancomycin) OR (TI dalbavancin OR AB dalbavancin) OR (TI linezolid OR AB linezolid) OR (TI rifampin OR AB rifampin) OR (TI ertapenem OR AB ertapenem) OR (TI imipenem OR AB imipenem) OR (TI meropenem OR AB meropenem) OR (TI ciprofloxacin OR AB ciprofloxacin) OR (TI beta-lactam OR AB beta-lactam) OR (TI beta-lactams OR AB beta-lactams) OR (TI cephalosporin OR AB cephalosporin) OR (TI cephalosporins OR AB cephalosporins) OR (TI carbapenem OR AB carbapenem) OR (TI carbapenems OR AB carbapenems) OR (TI aminoglycoside OR AB aminoglycoside) OR (TI aminoglycosides OR AB aminoglycosides) OR (TI fluoroquinolone OR AB fluoroquinolone) OR (TI fluoroquinolones OR AB fluoroquinolones))		
#3 *Combo Tx terms*	(MH "Drug Therapy, Combination") OR ((TI combination OR AB combination) OR (TI combinations OR AB combinations) OR (TI combined OR AB combined) OR (TI combining OR AB combining) OR (TI combo OR AB combo) OR (TI combos OR AB combos) OR (TI polytherap* OR AB polytherap*) OR (TI multimodal OR AB multimodal) OR (TI multi-modal OR AB multi-modal) OR (TI "add on" OR AB "add on") OR (TI add-on OR AB add-on) OR (TI co-treat* OR AB co-treat*) OR (TI cotreat* OR AB cotreat*) OR (TI co-therap* OR AB co-therap*) OR (TI cotherap* OR AB cotherap*) OR (TI co-admin* OR AB co-admin*) OR (TI coadmin* OR AB coadmin*) OR (TI synerg* OR AB synerg*)) OR ((TI plus) OR (TI multi) OR (TI multiple) OR (TI dual))	474317	487023
#4 combining	S1 AND S2 AND S3	301	306
#5	S4 NOT ((TI "case report") OR (TI "case reports") OR (TI "case study") OR (TI "case studies"))	264	269
#6	S5 NOT PT (Abstract OR Book OR Book Chapter OR Book Review OR Case Study OR Commentary OR Editorial OR Letter OR Masters Thesis OR Pamphlet OR Pamphlet Chapter OR Poetry)	148	164
Search update	Publication Date: 20220101-20241231	n/a	16

Supplemental Table S1. Detailed characteristics of included studies

Author, Year	Country	Study Design	Type of Pathogen	Number of Centres	Total Number of Patients	Combination Therapy Medications	Monotherapy Medications
Wilson 1957	USA	Retrospective Cohort	*Staphylococcus*	1	29	Different Combinations	Penicillin/Streptomycin/Sulfadiazine
Watanakunakorn 1977	USA	Retrospective Cohort	*Staphylococcus aureus*	NR	40	Penicillin/Nafcillin/Methicillin + Gentamicin	Penicillin G: 9<br>Methicillin: 8<br>Nafcillin: 4<br>Cephalothin: 3<br>Vancomycin: 1
Venturini 2024	Italy	Retrospective Cohort	methicillin-susceptible *Staphylococcus aureus*	NR	3	Oxacillin + Rifampin/Gentamicin<br>OR<br>Daptomycin + Oxacillin/Fosfomycin/Ceftaroline	Oxacillin/Daptomycin
Tuazon 1986	USA	Retrospective Cohort	*Streptococcus*	2	44	Penicillin + Aminoglycoside	Penicillin
Shah 2024 (*Pseudomonas*)	USA	Retrospective Cohort	*Pseudomonas aeruginosa*	11	48	Beta-lactam + aminoglycoside/fluoroquinolone<br>OR<br>other combinations	Cephalosporins/Penicillin
Shah 2024 (MSSA)	USA	Retrospective Cohort	methicillin-susceptible *Staphylococcus aureus*	5	98	Ertapenem/meropenem + standard treatment	Cefazolin/Oxacillin/Nafcillin
Shah 2023	USA	Retrospective Cohort	*Serratia* Species	12	75	Beta-lactam + aminoglycoside /fluoroquinolone<br>OR<br>Other combinations	Cefepime (n = 19)<br>Ceftazidime/ceftriaxone (n = 7)<br>Fluoroquinolones (n = 2)<br>Carbapenems (n = 8)<br>Piperacillin/tazobactam (n = 3)
Sexton 1998	USA	Randomized controlled trial	*Streptococcus*	9	51	Ceftriaxone + Gentamicin	Ceftriaxone
Schmit 1992	Europe	Prospective Cohort	*Enterococcus faecalis*	NR	18	Teicoplanin + Aminoglycoside	Teicoplanin
Rogers 2009	Australia	Retrospective Cohort	Methicillin resistant *Staphylococcus aureus*	1	27	[Vancomycin + Rifampicin + Fusidic acid (10)]<br>[Vancomycin + Rifampicin(6)]<br>[Linezolid + Rifampicin +Fusidic acid(2)]<br>[Other combinations(4)]	Vancomycin:3<br>Other:2
Ribera 1996	Spain	Randomized controlled trial	*Staphylococcus aureus*	1	74	Cloxacillin + Gentamicin	Cloxacillin
Peghin 2019	Italy	Prospective Cohort	*Enterococcus* spp.	1	16	Daptomycin + Ampicillin/Ceftaroline/Gentamicin	Daptomycin
Pallotto 2021[a]	Italy	Retrospective Cohort	*Streptococcus*	7	21	Daptomycin + Beta lactam/Rifampin	Daptomycin
Morpeth 2007	28 countries	Prospective Cohort	non-HACEK gram-negatives	61	48	Different Combinations	Different
Mogollon 2011	Spain	Retrospective Cohort	*Coxiella burnetii*	13	81	Doxycycline + one of below:<br>-Cotrimoxazole(19)<br>-Rifampicin(4)<br>-Quinolones (35)<br>-Changing Combination(7)<br>-Chloroquine(4)	Doxycycline

Malacoff 1979	USA	Retrospective Cohort	penicillin-sensitive Streptococci	2	68	Penicillin G/ Cephalothin/ Lincomycin/Vancomycin + Aminoglycoside (=>4days)	Penicillin G/ Cephalothin/ Lincomycin/Vancomycin + Aminoglycoside (=< 3 days)
Lorenz 2021	USA	Retrospective Cohort	non-HACEK Gram negative	1	60	Cephalosporin + Aminoglycoside (10)<br>Cephalosporin + Fluoroquinolone (9)<br>Carbapenem + Fluoroquinolone (5)<br>Penicillin + Fluoroquinolone (1)<br>Monobactam + Aminoglycoside (1)	Penicillin (3)<br>Cephalosporin (14)<br>Carbapenem (13)<br>Fluoroquinolone (4)
Levy 1991	France	Retrospective Cohort	*Coxiella burnetii*	9	30	Doxycycline + Rifampin/Quinolone<br>or<br>Rifampin + Sulfamethoxazole-trimethoprim	Doxycycline
Levine 1991	USA	Randomized controlled trial	Methicillin-resistant *Staphylococcus aureus*	1	42	Vancomycin + Rifampicin	Vancomycin
Leport 1989	France	Non-randomized trial	gram-positive cocci	multicenter	20	Teicoplanin + Aminoglycoside/ Fosfomycin/Rifampin	Teicoplanin
Korzeniowski 1982	USA	Randomized controlled trial	*Staphylococcus aureus*	NR	78	Nafcillin/Penicillin + Gentamicin	Nafcillin / Penicillin
Knoll 2007	USA	Retrospective Cohort	viridians group streptococci	1	29	different combinations	Ceftriaxone/ Ampicillin/ Vancomycin/ Penicillin G
Kaipainen 1957	Finland	Retrospective Cohort	NR	3	102	Penicillin + other (mainly Streptomycin)	Penicillin
Hughes 2009	USA	Retrospective Cohort	methicillin-susceptible *Staphylococcus aureus*	1	107	Oxacillin + Gentamicin	Oxacillin
Fadel 2009	USA	Retrospective Cohort	Streptococci and Staphylococci	1	105	Beta-lactam (penicillin or ceftriaxone) / Vancomycin + Aminoglycoside/rifampin	Beta-lactam (Penicillin / Ceftriaxone) / Vancomycin
Escrihuela-Vidal 2023	Spain	Prospective Cohort	Viridians group Streptococci and gallolyticus group Streptococci	40	899	Beta-lactam (Penicillin/Ampicillin/ Cephalosporin) + Aminoglycoside	Penicillin / Ampicillin / Ceftriaxone
Destache 2019	USA	Retrospective Cohort	gram positive	22	55	Ceftaroline Fosamil + another antibiotic	Ceftaroline Fosamil
Darras-Joly 1997	France	Retrospective Cohort	*Haemophilus*	24	42	Beta-lactam (Amoxicillin / Cephalosporin) + Aminoglycoside (29 patients [69%]);<br>Beta-lactam + Fluoroquinolone (7 [16.7%]);<br>Aminoglycoside + Fluoroquinolone (5 [11.9%]);<br>Beta-lactam + Aminoglycoside	Beta-lactam

						+ Fluoroquinolone (2 [4.8%]), Rifampin + Fluoroquinolone (3 [7.1%]); Rifampin + Fosfomycin (1 [2.4%])	
Danneels 2023	France	Retrospective Cohort	*Enterococcus faecalis*	14	279	Amoxicillin + Gentamicin/Ceftriaxone	Amoxicillin
Ceron 2014	Spain	Retrospective Cohort	*Enterococcus faecalis* and *faecium*	1	27	Ampicillin + Ceftriaxone	Daptomycin
Calderon Perra 2021	Spain	Prospective Cohort	non-HACEK Gram negatives	27	104	Beta-lactam + Aminoglycoside/ Quinolone or other combinations	Beta-lactam
Abrams 1979	USA	Randomized controlled trial	*Staphylococcus aureus*	NR	24	Gentamicin + penicillinase-resistant penicillin or a cephalosporin (Oxacillin:9 Oxacillin-penicillin:0 Oxacillin-cephalothin:3 Cephalothin:1)	Penicillinase-resistant Penicillin / a Cephalosporin (Oxacillin:7 Oxacillin-penicillin:2 Oxacillin-cephalothin:1 Cephalothin:2)

a: There were two groups in this study: Group A with 34 patients and Group B with 21 patients. Twenty-five patients received combination therapy and nine patients received monotherapy in group A. There were two deaths among group A, but it was unclear whether these deaths occurred in the monotherapy or combination therapy groups. n Group B, 20 patients received combination therapy and one patient received monotherapy, with no deaths reported. We included only Group B in our analysis as we had all the available mortality data specified based on the type of therapy.

Supplemental Table S2. The Newcastle-Ottawa Quality Assessment Scale determines risk of bias in observational studies through eight questions. For each study, the grades for the eight questions are shown below. Grades that receive a star are highlighted in green, while those that do not are highlighted in red. Based on the grades from each question in the Newcastle-Ottawa Scale, an overall risk of bias (high, medium, low) can be assigned.

Study	**Selection:** Representativeness of the exposed cohort	**Selection:** Selection of the non-exposed cohort	**Selection:** Ascertainment of exposure	**Selection:** Outcome of interest not present at start	**Comparability:** Study controls for confounders	**Outcome:** Assessment of outcome	**Outcome:** Follow-up long enough for outcomes to occur	**Outcome:** Adequacy of follow up of cohorts	Risk of Bias
Wilson 1957	a	a	a	a	c	d	b	d	Poor
Watanakunakorn 1977	a	a	a	a	c	b	a	a	Poor
Venturini 2024	b	a	a	a	b	d	a	d	Poor
Tuazon 1986	a	a	a	a	a, b	d	a	a	Good
Shah 2024 (Pseudomonas)	b	a	a	a	a, b	b	a	d	Good
Shah 2024 (MSSA)	b	a	a	a	a, b	b	a	d	Good
Shah 2023	b	a	a	a	a, b	b	a	d	Good
Schmit 1992	b	a	a	a	c	b	a	d	Poor
Rogers 2009	b	a	a	a	c	b	a	a	Poor
Peghin 2019	b	a	a	a	a, b	b	a	b	Good
Pallotto 2021	c	a	a	a	c	b	a	c	Poor
Morpeth 2007	c	a	a	a	c	b	a	a	Poor
Mogollon 2011	c	a	a	a	c	b	a	a	Poor
Malacoff 1979	b	a	a	a	b	b	a	d	Good
Lorenz 2021	b	a	a	a	c	b	a	d	Poor
Levy 1991	c	a	a	a	c	b	a	a	Poor
Leport 1989	b	a	a	a	b	a	a	a	Poor
Knoll 2007	b	a	a	a	c	b	a	a	Poor
Kaipainen 1957	d	a	a	a	c	b	a	d	Poor
Hughes 2009	b	a	a	a	a	b	a	d	Good
Fadel 2009	c	a	a	a	c	b	a	b	Poor
Escrihuela-Vidal 2023	b	a	b	a	c	b	a	a	Poor
Destache 2019	b	a	a	a	c	b	a	c	Poor
Darras-Joly 1997	b	a	b	a	c	e	a	a	Poor
Danneels 2023	b	a	a	a	a, b	b	a	d	Good
Ceron 2014	b	a	a	a	b	b	b	a	Good
Calderon Parra 2021	a	a	a	a	b	b	a	d	Good

Supplemental Table S3. Influence analysis of the meta-analysis for studies conducted in Europe.
Omission of the study performed by Escrihuela-Vidal et al. resulted in a non-significant result (p=0.19).

	**OR**	**95%-CI**	**p-value**	**tau^2**	**tau**	***I*^2**
Omitting Calderon Parra 2021	0.6386	[0.4800; 0.8498]	0.0021	0.0000	0.0000	0.0%
Omitting Ceron 2014	0.6574	[0.4986; 0.8670]	0.0030	0.0000	0.0000	0.0%
Omitting Danneels 2023	0.6724	[0.5082; 0.8898]	0.0055	0.0000	0.0000	1.0%
Omitting Darras-Joly 1997	0.6778	[0.5145; 0.8929]	0.0057	0.0000	0.0000	0.0%
Omitting Escrihuela-Vidal 2023	0.7063	[0.4203; 1.1869]	0.1893	0.0684	0.2615	0.5%
Omitting Kaipainen 1957	0.6945	[0.5211; 0.9257]	0.0129	0.0000	0.0000	0.0%
Omitting Leport 1989	0.6740	[0.5117; 0.8879]	0.0050	0.0000	0.0000	1.1%
Omitting Levy 1991	0.7064	[0.5350; 0.9325]	0.0142	0.0000	0.0000	0.0%
Omitting Mogollon 2011	0.6672	[0.5050; 0.8816]	0.0044	0.0000	0.0000	0.0%
Omitting Pallotto 2021	0.6738	[0.5119; 0.8868]	0.0048	0.0000	0.0000	0.0%
Omitting Peghin 2019	0.6699	[0.5086; 0.8825]	0.0044	0.0000	0.0000	0.0%
Omitting Ribera 1996	0.6637	[0.5034; 0.8751]	0.0037	0.0000	0.0000	0.0%
Omitting Schmit 1992	0.6786	[0.5151; 0.8939]	0.0058	0.0000	0.0000	0.0%
Omitting Venturini 2024	0.6751	[0.5111; 0.8917]	0.0057	0.0000	0.0000	1.0%
**Pooled estimate**	**0.6738**	**[0.5119; 0.8868]**	**0.0048**	**0.0000**	**0.0000**	**0.0%**

Supplemental Figure S1. Rob2 Risk of Bias assessment for randomized control trials

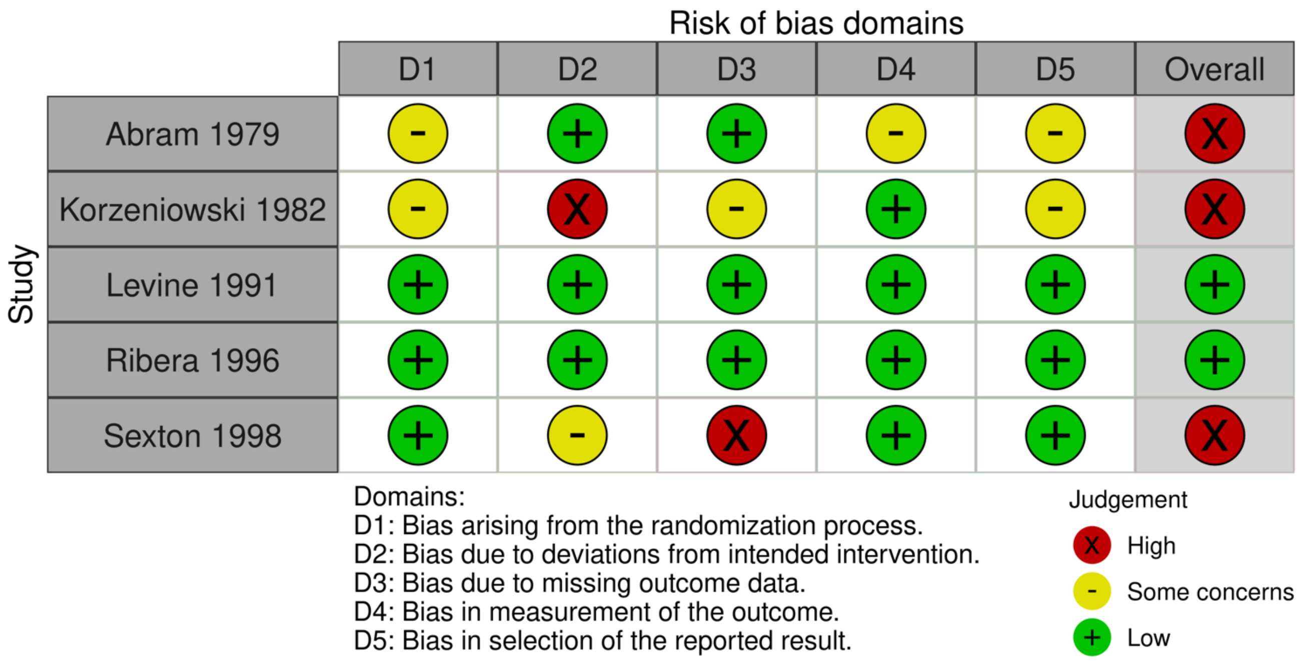

Supplemental Figure S2. A funnel plot with included studies did not reveal evidence of publication bias (Egger's test p-value = 0.11).

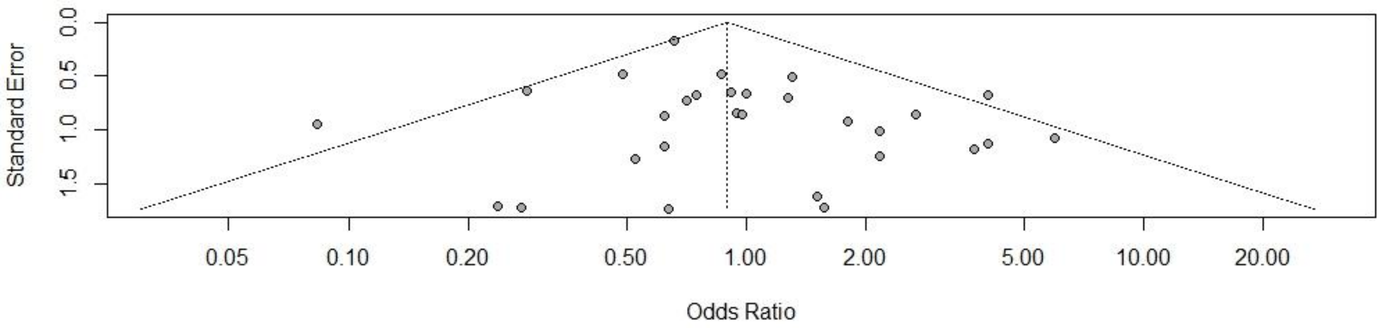

Supplemental Figure S3. Forest plot of in-hospital mortality in patients with infective endocarditis treated with monotherapy versus combination therapy.

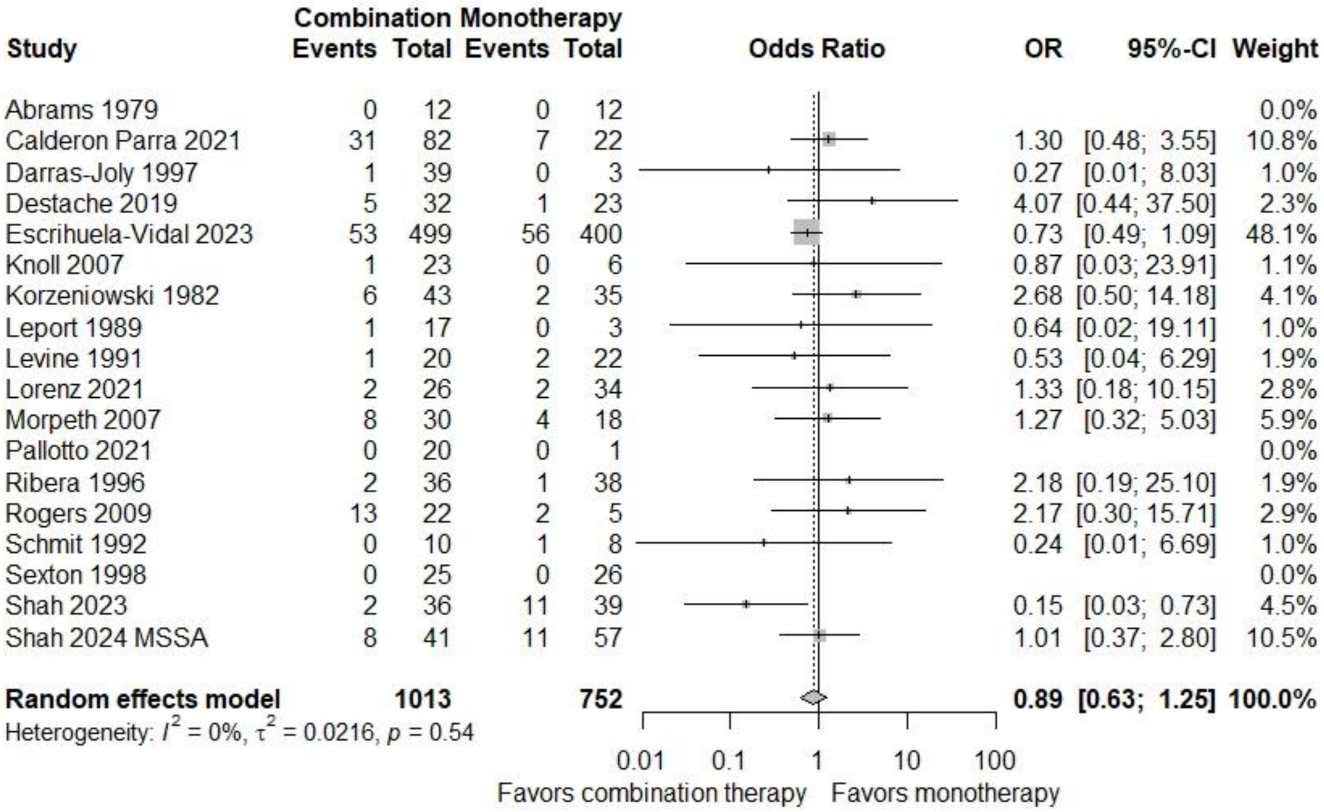

Supplemental Figure S4. Forest plot of 30-day mortality in patients with infective endocarditis treated with monotherapy versus combination therapy.

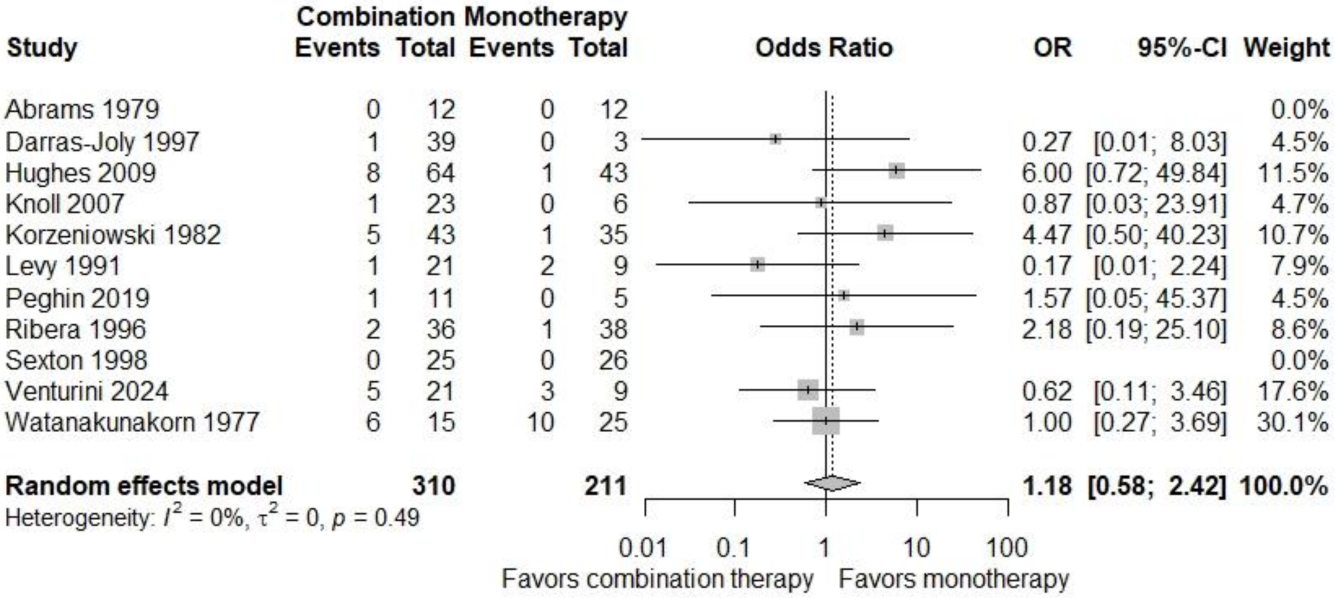

Supplement Figure S5. **Forest plot of 1 year mortality in patients with infective endocarditis treated with monotherapy versus combination therapy.**

Study	Combination Events	Combination Total	Monotherapy Events	Monotherapy Total	OR	95%-CI	Weight
Calderon Parra 2021	36	82	8	22	1.37	[0.52; 3.62]	16.8%
Danneels 2023	68	260	3	9	0.71	[0.17; 2.91]	9.6%
Escrihuela-Vidal 2023	80	499	90	400	0.66	[0.47; 0.92]	41.1%
Kaipainen 1957	29	73	21	29	0.25	[0.10; 0.64]	17.5%
Levy 1991	1	21	2	9	0.17	[0.01; 2.24]	3.4%
Malacoff 1979	1	19	4	49	0.62	[0.07; 5.98]	4.2%
Mogollon 2011	10	68	2	13	0.95	[0.18; 4.93]	7.4%
**Random effects model**		**1022**		**531**	**0.62**	**[0.38; 1.01]**	**100.0%**

Heterogeneity: $I^2 = 19\%$, $\tau^2 = 0.1204$, $p = 0.28$

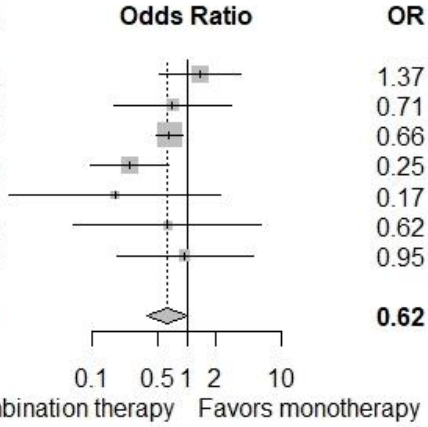

Supplemental Figure S6. **Forest plot of overall mortality in patients with gram positive infective endocarditis treated with monotherapy versus combination therapy.** The primary mortality endpoint (e.g. in-hospital mortality, 30-day mortality, etc.) for each study is represented here.

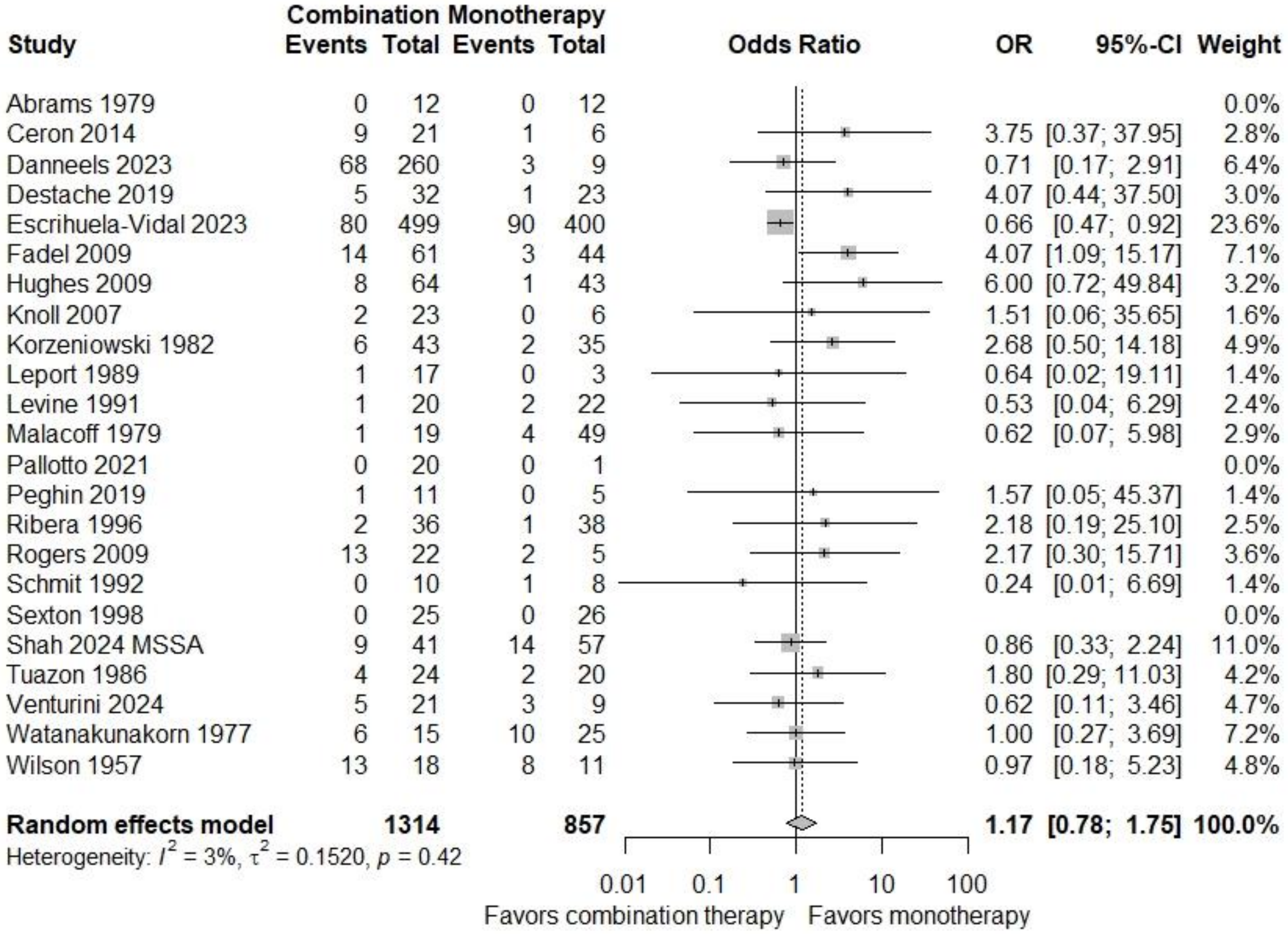

Supplemental Figure S7. **Forest plot of overall mortality in patients with gram negative infective endocarditis treated with monotherapy versus combination therapy.** The primary mortality endpoint (e.g. in-hospital mortality, 30-day mortality, etc.) for each study is represented here.

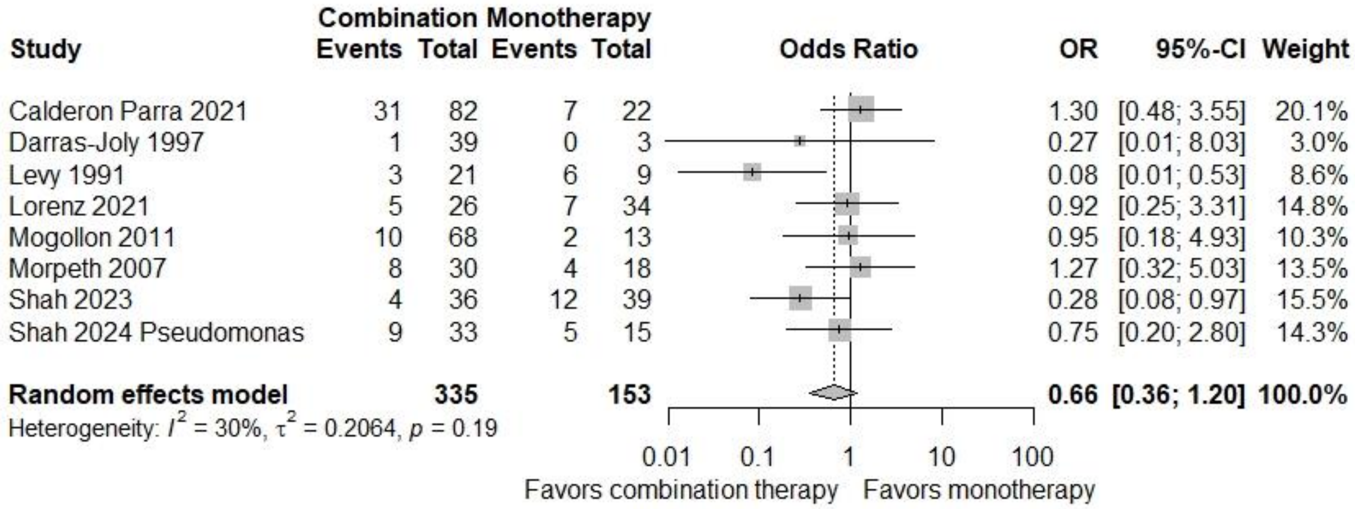

Supplemental Figure S8. Forest plot of overall mortality in patients with infective endocarditis treated with monotherapy versus combination therapy for studies conducted in United states. The primary mortality endpoint (e.g. in-hospital mortality, 30-day mortality, etc.) for each study is represented here.

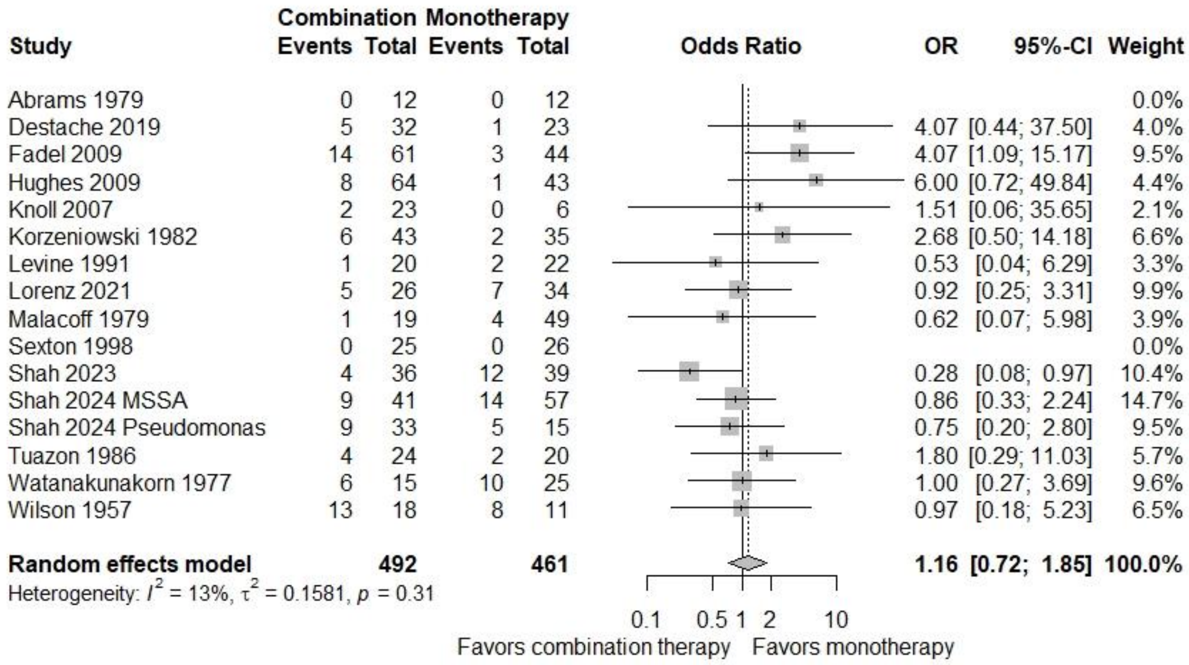

Supplemental Figure S9. **Forest plot of overall mortality in patients with infective endocarditis treated with monotherapy versus combination therapy for observational studies.** The primary mortality endpoint (e.g. in-hospital mortality, 30-day mortality, etc.) for each study is represented here.

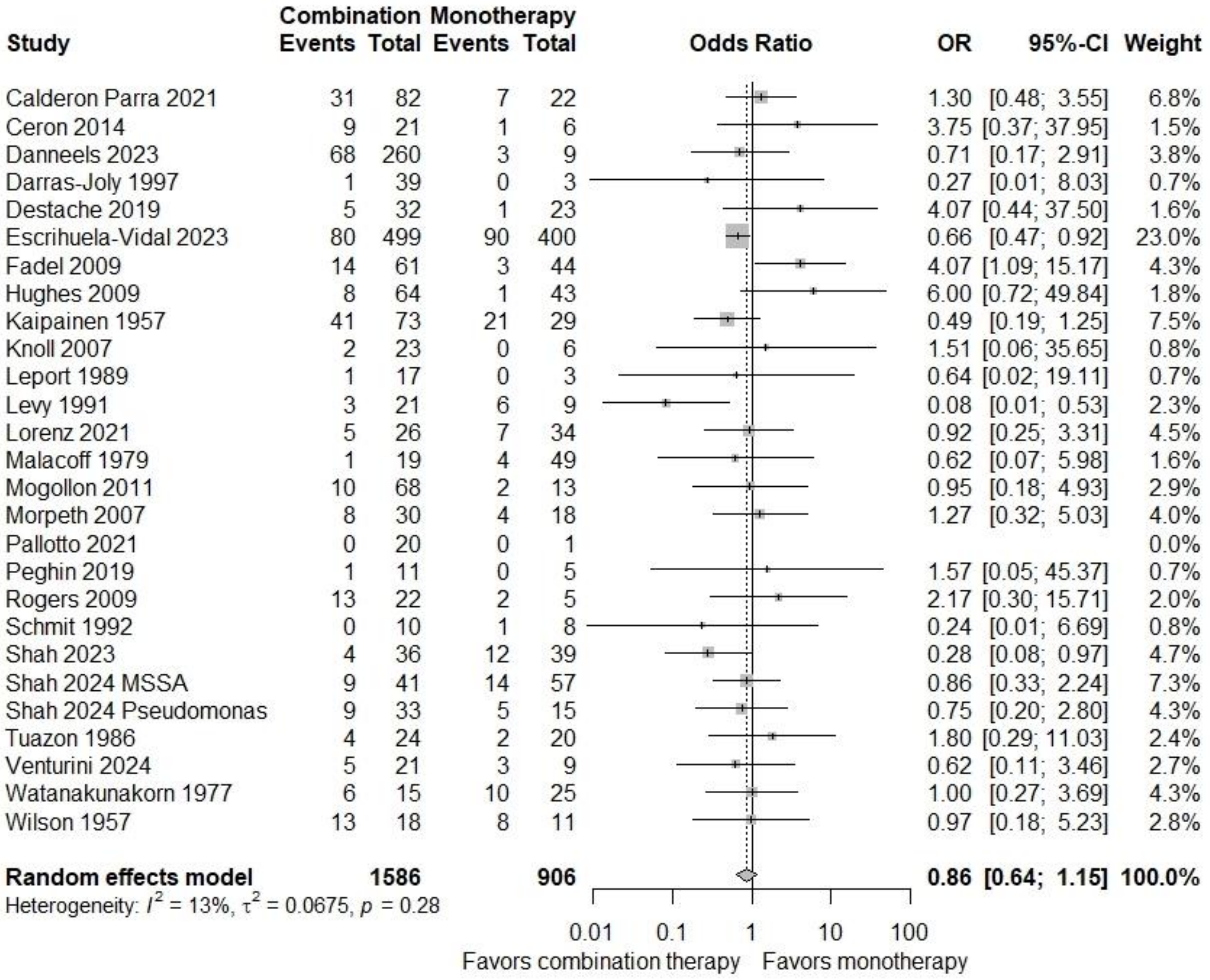

Supplemental Figure S10. **Forest plot of overall mortality in patients with infective endocarditis treated with monotherapy versus combination therapy for randomized controlled trials.** The primary mortality endpoint (e.g. in-hospital mortality, 30-day mortality, etc.) for each study is represented here.

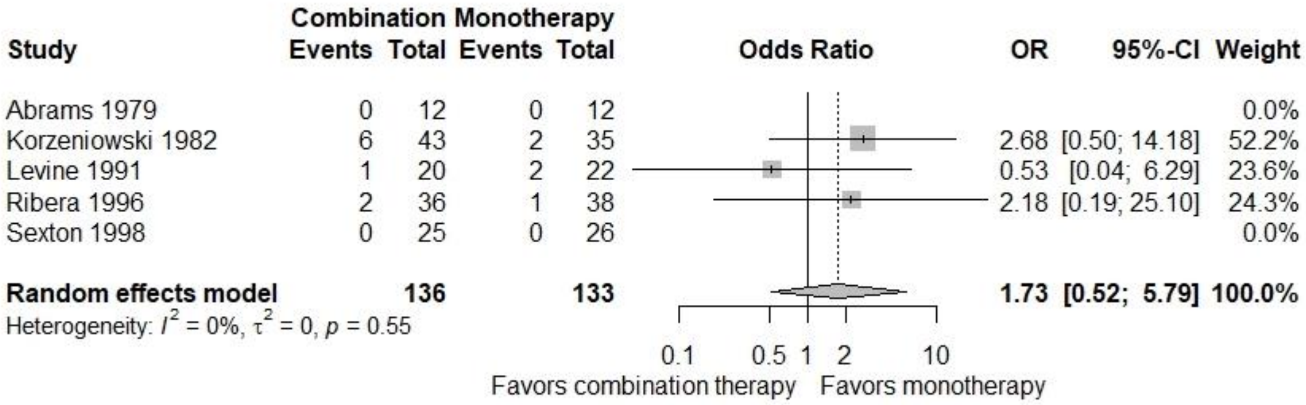

Supplemental Figure S11. **Forest plot of overall mortality in patients with infective endocarditis treated with monotherapy versus combination therapy for studies published before 2000.** The primary mortality endpoint (e.g. in-hospital mortality, 30-day mortality, etc.) for each study is represented here.

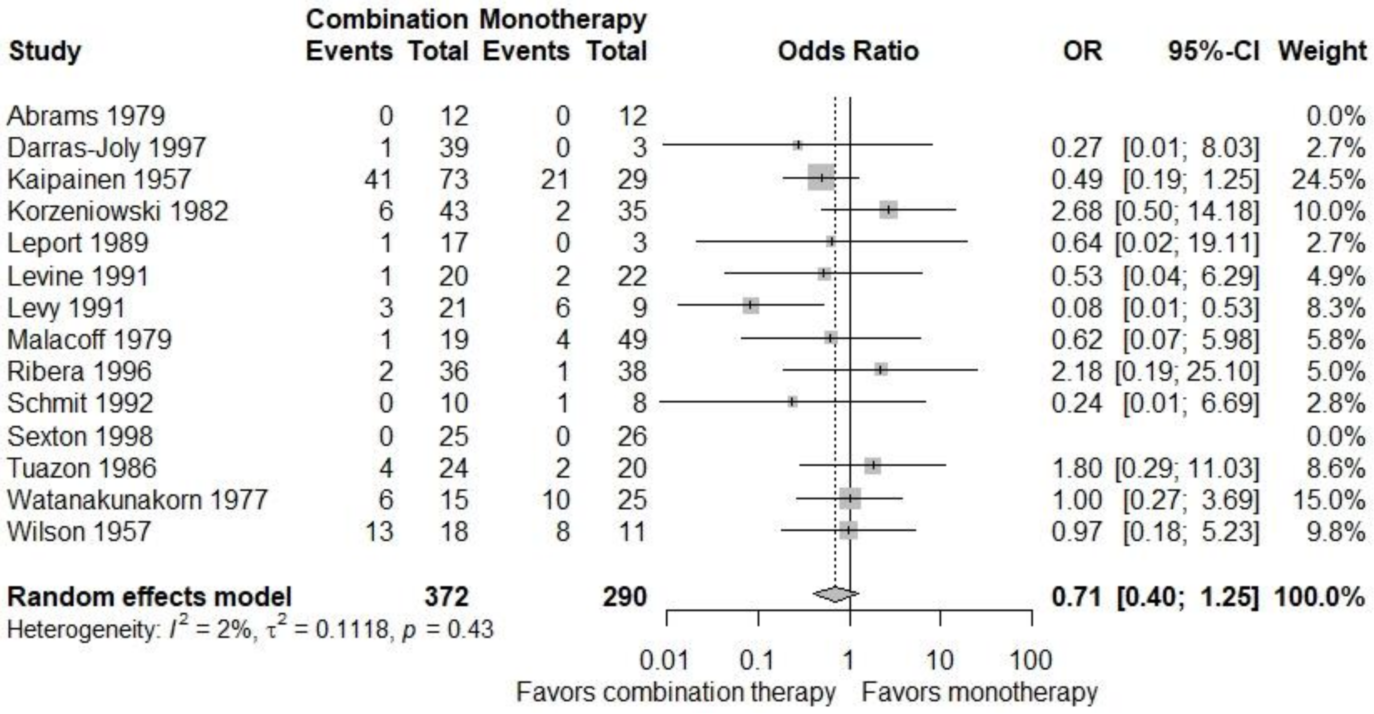

Supplemental Figure S12. **Forest plot of overall mortality in patients with infective endocarditis treated with monotherapy versus combination therapy for studies published after 2000.** The primary mortality endpoint (e.g. in-hospital mortality, 30-day mortality, etc.) for each study is represented here.

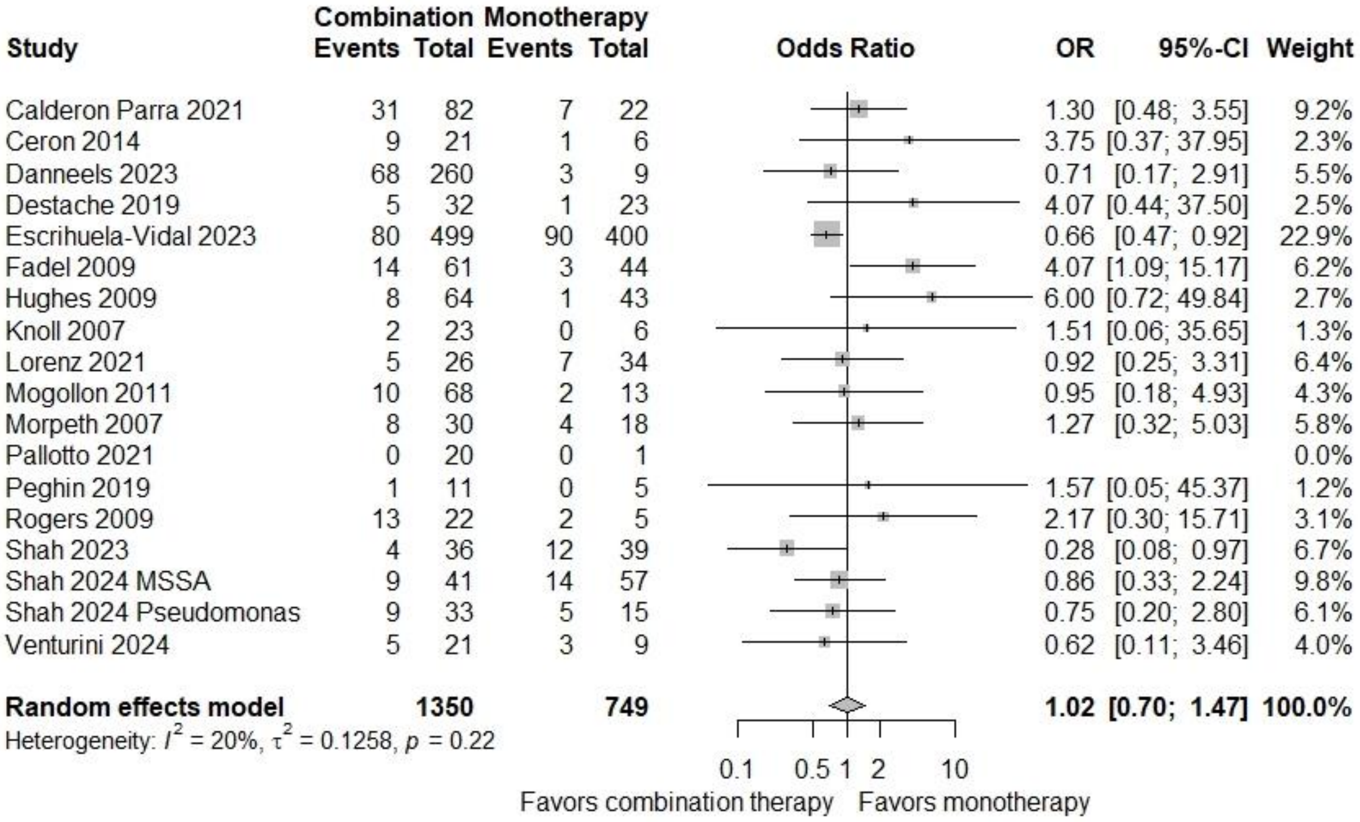

Supplement: Supplementary file 1 [file microorganisms-12-02226-s001.zip › microorganisms-3264695-supplementary.pdf]
